# Urinary Exosomes in Nephrology: A New Frontier for Diagnosis and Prognosis of Kidney Diseases

**DOI:** 10.3390/ijms26178679

**Published:** 2025-09-05

**Authors:** Costanza Gaudio, Emanuele D’Arpino, Simone Stefani, Filippo Maria Fani, Giuseppina Rosso, Elio Di Marcantonio, Paola Becherelli, Gianmarco Caselli, Chiara Merciai, Laura Fortunato, Nicoletta Scopetani, Alberto Rosati

**Affiliations:** Unit of Nephrology, San Giovanni di Dio Hospital, Department of Medical Specialties, Azienda USL Toscana Centro, 50143 Florence, Italy; costanza.gaudio@uslcentro.toscana.it (C.G.); emanuele.darpino@unifi.it (E.D.); simone.stefani@unifi.it (S.S.); filippomaria.fani@uslcentro.toscana.it (F.M.F.); giuseppina.rosso@uslcentro.toscana.it (G.R.); elio.dimarcantonio@uslcentro.toscana.it (E.D.M.); paola.becherelli@uslcentro.toscana.it (P.B.); gianmarco.caselli@uslcentro.toscana.it (G.C.); chiara.merciai@uslcentro.toscana.it (C.M.); laura.fortunato@uslcentro.toscana.it (L.F.); nicoletta.scopetani@uslcentro.toscana.it (N.S.)

**Keywords:** urinary exosomes, membranous nephropathy, diabetic nephropathy, IgAN, MGRS, FSGS, MCD, kidney transplant, mass spectrometry

## Abstract

Exosomes are nanosized vesicles that carry intracellular mediators and their abundance in urine opens new and intriguing possibilities in nephrology since they provide a non-invasive insight into kidney diseases. The aim of this review is to examine the main applications of urinary exosomes in nephropathies. Urinary exosomes are isolated through ultrafiltration, ultracentrifugation, precipitation, and immunoaffinity chromatography. After isolation they are characterized through Western blotting, flow cytometry, and, more recently, with mass spectrometry. Through the analysis of urinary exosomes, it has been possible to distinguish patients with IgA nephropathy from healthy controls. Different profiles of expression have been identified between patients with MCD and FSGS. A distinct exosomal composition has been discovered in patients with lupus nephropathy when compared to those without renal involvement. Significant findings have been reported also in patients with monoclonal gammopathy of renal significance, allowing a differential diagnosis between LCDD and amyloidosis. Among kidney transplant recipients, the analysis of urinary exosomes highlighted differences between antibody-mediated rejection and cell-mediated rejection. Urinary exosomes are new non-invasive, promising biomarkers and potential therapeutic options that have already shown interesting results in the nephrological field. Further studies are needed to harness their potential and diffusion.

## 1. Introduction

The Kidney Disease: Improving Global Outcomes (KDIGO) guidelines advocate the integration of Cause (C), Glomerular Filtration Rate (GFR, G), and Albuminuria (A)—collectively known as CGA staging—as a framework to guide specialist referral, general clinical management, and the initiation of diagnostic or therapeutic interventions [1]. The etiological classification of CKD is emphasized due to its pivotal role in prognostication and the selection of disease-specific therapies.

Despite this, routine CKD risk stratification often relies on estimated GFR and albuminuria alone, without sufficient consideration of the underlying cause of kidney disease [2]. Accurate etiologic diagnosis remains essential for therapeutic decision-making, risk stratification, prediction of post-transplant recurrence, and evaluation of hereditary transmission. Renal biopsy is frequently central to this process.

Over the past five decades, renal pathology has evolved substantially, and by the early 2000s, diagnostic capability had surpassed pathophysiological understanding. Today, renal biopsy is considered indispensable in nephrology; however, globally endorsed guidelines on its indications are still lacking [3]. Nonetheless, biopsy has inherent limitations—it provides a static, focal representation of renal architecture, potentially failing to capture diffuse pathology. Hence, pathological findings should be interpreted in conjunction with the overall clinical picture to guide optimal decision-making.

Additional limitations stem from inter-observer variability in histological interpretation. A pivotal study revealed significant discrepancies among international renal pathologists evaluating the same allograft biopsy slides [4]. Moreover, procedural risks—particularly bleeding—must be considered. In a meta-analysis, the incidence of macroscopic hematuria post-biopsy was 3.5%, and transfusion was required in 0.9% of cases [5]. These risks are exacerbated in advanced CKD due to cortical thinning and increased vascular fragility. Finally, it is important to note that certain clinical conditions may constitute relative or, in some cases, absolute contraindications to performing the examination. These include renal cysts, uncorrectable coagulopathies, severe obesity, congenital, or acquired anatomical abnormalities such as horseshoe kidney. Additionally, a solitary functioning kidney, or uncooperative patients must be taken into account. In such cases, the potential risks of hemorrhagic complications or permanent organ damage may be deemed to outweigh the diagnostic benefits.

In recent years, genetic testing has emerged as a powerful diagnostic adjunct—and in some contexts, a potential alternative—to biopsy. Studies indicate that monogenic disorders account for 30–50% of pediatric CKD and 10–20% of adult cases [6,7].

However, the European registry reports that the percentage of ESRD patients with a diagnosis of unknown renal disease is still very high, around 37%. Therefore, in over 1/3 of patients, current diagnostic tools failed to establish a correct diagnosis of renal disease [8].

Therefore, the accurate diagnosis of primary renal disease is a fundamental objective for guiding treatment, risk stratification for disease progression, post-transplant relapses, and estimating hereditary transmission in genetic diseases.

This underscores the necessity for integrating genetic and other non-invasive investigations that are sufficiently sensitive and specific to provide accurate diagnoses across the spectrum of renal diseases.

Exosomes are nanosized vesicles measuring between 40 and 120 nm. They are characterized by a phospholipid membrane since they derive from the exocytosis of late endosomes through the plasma membrane [9].

Exosomes carry a range of intracellular mediators such as glycoproteins, nuclear proteins, miRNA and cytoplasmic proteins and play a pivotal role in many physiological processes, including paracrine signaling and immune regulation. Their membranes are enriched with flotillin and annexins and antigens such as CD9 and CD63 which facilitate their detection and isolation [10].

Their special coated structure determines the stability of the mediators they vehicle, allowing their detection even at particularly low concentrations [11]. Therefore, exosomes have been isolated from various biological fluids, including blood, cerebrospinal fluid, and pleural effusion, establishing their potential in the oncological field and expanding the concept of liquid biopsy.

However, their abundance in urine unlocks new and intriguing possibilities in the field of nephrology since they provide an accessible and non-invasive insight into kidney diseases. To date, almost 3000 different urinary exosomes, deriving from podocytes, tubular epithelial cells but also prostate, bladder, and residing immune cells have been identified thanks to the newest mass spectrometry techniques [12]. The molecular composition of exosomes is highly dynamic and reflects both the identity of the originating cell and the specific functional roles they are destined to fulfill within recipient cells. This variability endows exosomes with the capacity to convey detailed information about the physiological or pathological processes occurring within a given cellular population [13]. As such, exosomes represent a molecular fingerprint, shaped by the cellular context, and hold considerable potential as biomarkers. Indeed, the analysis of urinary exosome expression in patients with major nephropathies has unveiled promising avenues for non-invasive differential diagnosis and prognostic stratification. This positions urinary exosomes as valuable biomarkers within the emerging field of liquid biopsy, a diagnostic approach that utilizes biological fluids, including blood and urine, to evaluate and identify the presence of disease. Owing to their nanoscale dimensions, their inherent capacity to cross biological membranes, and their critical function in intercellular communication and molecular signaling, exosomes also present considerable therapeutic potential [14]. They can be harnessed as vectors for the targeted delivery of pharmacological agents, nucleic acids, or therapeutic genes. Furthermore, the possibility of engineering exosomes to enhance their specificity and affinity toward selected cellular targets opens new frontiers for precision medicine in nephrology.

The aim of this review is to provide insight into the main applications of urinary exosomes in the most clinically relevant nephropathies.

## 2. Urinary Exosomes Isolation and Characterization

To affirm their potential as non-invasive biomarkers and therapeutic vehicles, efficient isolation of urinary exosomes is mandatory, as their accurate characterization—often performed with downstream techniques such as proteomics—relies heavily on the integrity and purity of the isolated exosomes.

In the following section, we will provide an overview of the main methods currently employed for the isolation and characterization of urinary exosomes, underlining their respective advantages and limitations.

## 3. Isolation Methods

**Precipitation** uses the hydrophilic properties of polymers, such as polyethylene glycol (PEG), to induce the aggregation of vesicles by reducing their solubility in the aqueous phase. However, the potential contamination with residual polymers is difficult to eliminate and can interfere with downstream analyses, including proteomic profiling and functional assays [15].

**Ultracentrifugation**, considered a gold standard in many protocols, separates particles based on their density using extremely high centrifugal forces. It allows the processing of large sample volumes and provides relatively pure vesicular fractions when differential and density gradient centrifugation steps are appropriately combined. However, ultracentrifugation requires expensive and sophisticated equipment and is highly time-consuming, often requiring a gradual increase in centrifuge speed [16]. Moreover, the high shear forces generated during prolonged centrifugation may damage or deform delicate vesicle structures, potentially affecting their characterization [17].

**Ultrafiltration** represents an alternative approach in which exosomes are physically retained by membrane filters with specific pore sizes, often made of polysulfone [18]. This technique is capable of isolating exosomes from relatively large volumes of biofluids. However, it is associated with the risk of vesicle loss due to membrane adsorption and size exclusion biases. Furthermore, ultrafiltration-derived preparations may be incompatible with certain downstream analyses, given the potential contamination from filter materials or retention of other macromolecules [19].

**Immunoaffinity chromatography** is a highly selective method based on the interaction between surface markers on exosomes and target antibodies located on a chromatographic matrix. This technique allows the isolation of exosomes with a particular surface antigen profile (e.g., CD9, CD63, CD81), with high specificity. However, its use is often limited by high cost and its sensitivity to variations in pH, ionic strength, and buffer composition that can significantly affect their binding efficiency and reproducibility [20].

In light of the limitations of the overexposed methods, research has focused on validating new alternative approaches such as EXODUS (exosome detection via the ultrafast isolation system) and asymmetric flow field-flow fractionation (AF4). The EXODUS platform [21] is a rapid and label-free isolation system with improved sensitivity and minimal sample preparation; however, its substantial cost and the need for an automated workstation limit its diffusion to research environments with significant funding. AF4 [22], conversely, is a non-invasive, size-based separation technique that preserves vesicle integrity and allows for high-resolution fractionation of heterogeneous extracellular vesicles.

Once exosomes have been successfully isolated, their characterization is needed to evaluate their identity, to assess their purity, and to analyze their molecular cargo.

According to the guidelines established by the International Society for Extracellular Vesicles (ISEV), the detection of specific surface markers such as tetraspanins (CD9, CD63, CD81), integrins, TSG101, and other exosome-associated proteins is required to perform a solid exosome characterization.

Techniques commonly used for this purpose include Western blotting, which performs a separation of proteins in a biological sample through electrophoresis and subsequent detection using specific antibodies; flow cytometry combined with bead coupling, which analyzes the signal particles emit when a beam of light passes through them; and mass spectrometry, which, after ionizing the molecules, proceeds to characterize them based on their charge-to-mass ratio with high sensitivity and specificity.

These methods, often used in combination, help ensure that the isolated vesicles meet the criteria for exosomes, enabling their subsequent use in the diagnostic and therapeutic field [23].

## 4. Discussion

Exosomes are primarily defined by their peculiar and highly regulated endosomal biogenesis pathway [9,11,24]. This process distinguishes them from other extracellular vesicles (EVs), like those that bud directly from the plasma membrane (e.g., microvesicles), and is essential for understanding their diverse roles in intercellular communication [9].

**Figure 1:**
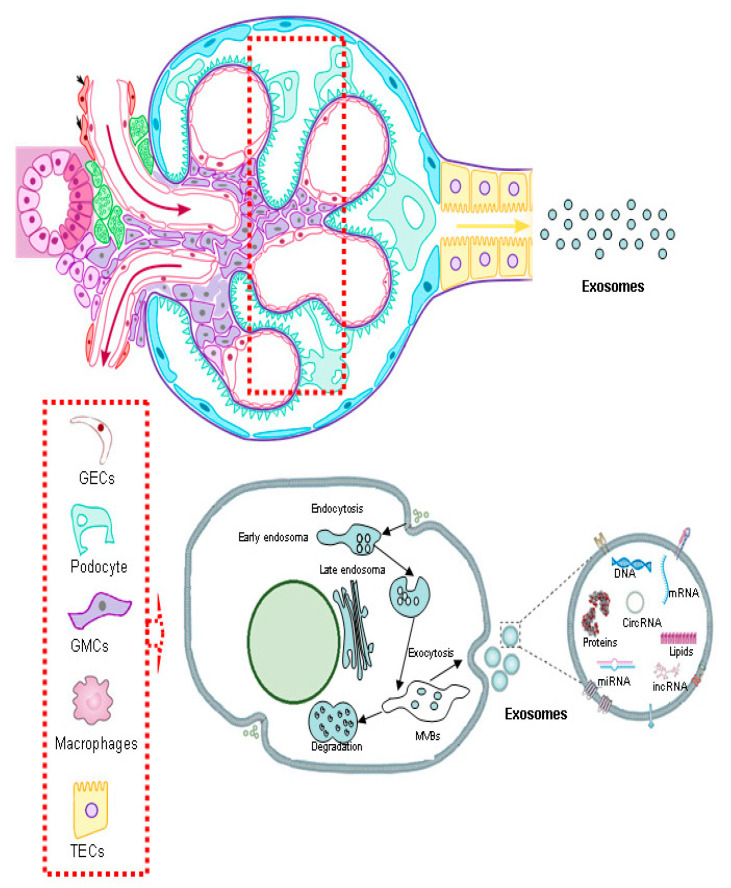
Formation of urinary exosomes in the kidney and their molecular content. They originate within multivesicular bodies (MVBs) and are released into the urinary lumen through fusion with the plasma membrane. Urinary exosomes carry biomolecules, including proteins, lipids, messenger RNAs (mRNAs), microRNAs (miRNAs), and DNA, reflecting the physiological or pathological state of their cells of origin. (CircRNA) Circular ribonucleic acid; (lncRNA) Long Non-Coding RNA; (GECs) glomerular endothelial cells; (GMCs) glomerular mesangial cells; (TECs) tubular epithelial cells.

Exosome formation initiates with endocytosis, (Figure 1) a widespread cellular phenomenon during which portions of the plasma membrane invaginate to form early endosomes [11]. These early endosomes then undergo a maturation process involving Golgi complex, which progressively transforms them into late endosomes. Inside these late endosomes, the crucial step of exosome biogenesis unfolds as their limiting membrane begins to invaginate inward [11,14]. This secondary budding mechanism leads to the encapsulation of cytoplasmic components and specific membrane proteins, generating small intraluminal vesicles (ILVs) within the lumen of the endosome. Once filled with ILVs, the late endosome is then defined as a multivesicular body (MVB) [10,11,14,24].

The precise orchestration of ILV formation is predominantly mediated by the Endosomal Sorting Complex Required for Transport (ESCRT) machinery [11,25]. This system is a multiprotein complex built of distinct subunits (ESCRT-0, ESCRT-I, ESCRT-II, ESCRT-III) and the ATPase VPS4, which act in a sequential and cooperative manner [11,24]. ESCRT-0 begins the process by recognizing and clustering ubiquitinated cargo on the endosomal membrane, marking them for inward sorting. Subsequently, ESCRT-I and ESCRT-II complexes facilitate initial membrane curvature and recruit ESCRT-III polymers, which then mediate the final steps of membrane invagination and scission, allowing the ILVs to bud off into the MVB lumen [11,25]. The ATPase VPS4 is essential for dismantling and recycling the ESCRT components, ensuring the machinery’s continuous availability for forthcoming budding events [11,25]. While the ESCRT pathway is the main driver, some studies indicate the existence of ESCRT-independent mechanisms, suggesting alternative methods for ILV formation [11].

After their formation, MVBs can fuse with lysosomes, leading to the degradation of their contents and serving as a mechanism for disposing of cellular waste [11,24]. Alternatively, and critically for exosome release, MVBs can migrate to the cell periphery and fuse with the plasma membrane. This fusion event results in the controlled release of the ILVs, which are now fully formed exosomes, into the extracellular space. Active secretion is the defining characteristic of exosome biology, enabling them to precisely deliver their molecular cargo to recipient cells.

During this thorough biogenesis, exosomes selectively package specific proteins, lipids, and nucleic acids (including miRNAs and mRNAs), whose composition reflects the physiological state of the parent cell [10,11,13,24]. This precise cargo loading, intrinsically linked to their formation pathway, gives exosomes the ability to carry specific molecular signals, making them essential communicators in various physiological processes [9,10,14]. Consequently, their highly regulated formation is pivotal for their role in orchestrating cellular communication and maintaining biological homeostasis.

## 5. Urinary Exosomes in Kidney Diseases

Urinary exosome analysis could represent a novel and complementary tool for clinicians in the diagnostic and prognostic evaluation of kidney diseases, potentially offering a non-invasive window into kidney pathology. The molecular cargo of these vesicles provides a dynamic and integrative portrait of kidney pathophysiology by reflecting the physiological or pathological state of their parent cells. In this context, urinary exosomes offer a highly promising non-invasive alternative to traditional tools that capture real-time molecular alterations across the entire nephron mass. According to our current understanding of molecular physiology, they are not simply cellular debris, but functionally active participants in intra- and intercellular communication, modulating processes such as immune cell activation, fibrosis, oxidative stress and apoptosis, all of which are central concepts in the pathophysiology of most kidney diseases.

The aforementioned technological advances have enabled detailed profiling of exosomal cargo, revealing disease-specific expression signatures that can be quantitatively assessed and monitored longitudinally. Moreover, urinary exosomes are uniquely suited for kidney-related biomarker discovery due to the direct anatomical proximity of the kidney to the urinary tract, minimizing systemic dilution and maximizing signal specificity.

From a more clinical and pragmatic point of view, the possibility to detect early any subclinical change in renal pathology via urinary exosome profiling foreshadows new prospectives for timely diagnosis, prognostic stratification, treatment individualization, and therapeutic monitoring.

This is particularly relevant in conditions where early intervention significantly modifies the disease course, such as diabetic nephropathy, lupus nephritis, and antibody-mediated rejection in kidney transplantation. Moreover, in conditions where serum biomarkers or clinical features are non-specific or ambiguous, exosomal signatures may offer an additional layer of diagnostic resolution. The reproducibility of certain exosomal biomarkers across patient cohorts may further support their potential for clinical translation. At the same time, the heterogeneity of techniques underscores the need for standardization and validation in larger, multicenter studies.

In this section, we discuss the identification and significance of urinary exosomal biomarkers in common nephropathies, including lupus nephritis, diabetic nephropathy, and rejection after kidney transplant (Table 1).

- IgA Nephropathy

Immunoglobulin A (IgA) nephropathy (IgAN) is a form of mesangial proliferative glomerulonephritis characterized by diffuse mesangial deposition of IgA and it is the most common cause of primary glomerulonephritis worldwide, with a peak incidence occurring in the second and third decades of life. No clinical pattern is pathognomonic of IgAN, its clinical presentation ranging from asymptomatic haematuria to acute kidney injury, therefore diagnosis cannot be made without a kidney biopsy [37].

Persistent isolated microhaematuria is indeed one of the most common indications for kidney biopsy, aimed at the early detection of IgAN and its differentiation from other conditions with a significantly more favorable prognosis, such as thin basement membrane nephropathy (TBMN) or even from healthy individuals.

Moon et al. [26] analyzed with mass spectrometry urinary exosomes from patients with IgAN, TBMN, and healthy controls, resulting in the characterization of 1877 proteins. Their findings demonstrated that patients with IgAN exhibited increased expression of aminopeptidase N, vasorin precursor, α-1-antitrypsin, and ceruloplasmin. Another study from Min QH et al. [27] compared the urinary exosomes miRNA profile between eighteen patients with IgA nephropathy and healthy controls and found that miR-29c, miR-146a, and miR-205 were more expressed in the affected individuals, highlighting their role as a potential new diagnostic marker of the disease.

- Podocytopathies

Podocytopathies are kidney diseases in which direct or indirect podocyte injury drives proteinuria or nephrotic syndrome. Despite the tendency for clinical manifestations to be uniform, there is considerable heterogeneity within the broad spectrum of podocytopathies, both in terms of etiopathogenetic distinctions between immune-mediated, genetic, and maladaptive causes, and in terms of histopathological patterns of damage. The two main patterns of damage, minimal change disease (MCD) and focal segmental glomerulosclerosis (FSGS), are characterized by significantly different data in terms of prognosis [38].

Differential diagnosis between MCD and FSGS could be tricky when based on clinical data. Ramezani et al. [28] were able to distinguish these two entities through the detection of up-regulation of miR-1225-5p found in the urinary exosomes of patients with MCD compared with those with FSGS. Interestingly, they observed that this peculiar expression in terms of miRNA did not change during time, and was not influenced by sex or age, since patients were matched to controls for these parameters.

- Membranous nephropathy

Membranous nephropathy is an immune complex-mediated disease characterized by the deposition of IgG and complement components predominantly on the subepithelial surface of the glomerular capillary wall, beneath podocytes. The resulting podocyte injury has been demonstrated to increase glomerular permeability. This, in turn, has been shown to result in proteinuria and, in certain cases, nephrotic syndrome [39].

The discovery of antibodies directed at the phospholipase A2 M-type phospholipase receptor (anti PLA2R) and at the thrombospondin type 1 domain-containing 7A (anti THSD7A) has deeply changed the diagnostic approach in membranous nephropathy, since kidney biopsy is no longer required to establish a diagnosis of membranous nephropathy in patients with positive autoantibodies and nephrotic syndrome. Although research has led to the discovery of novel autoantibodies and the development of techniques to detect them in histological samples, a significant number of idiopathic membranous nephropathy still tests negative for known autoantibodies both in serum and in tissue. As a result, kidney biopsy, with its implied risks, remains a necessary diagnostic tool in such cases.

The possibility of a non-invasive diagnosis of membranous nephropathy also in anti PLA2R negative patients through the analysis of urinary exosomes could become a reality, as reported by Wang et al. [29] who observed an overexpression of Alix, CD63, and TSG101 in urinary exosomes of patients with membranous nephropathy compared to healthy controls. Furthermore, data in the literature suggest that the analysis of these microvesicles may also provide prognostic information in PLA2R-positive patients: lower levels of Nrf2 or NLRP3 seem to correlate with a better response to treatment [29] and could therefore represent a future prognostic marker.

- Lupus Nephritis

In patients with systemic lupus erythematosus (SLE), determining whether a decline in renal function is due to the onset of lupus nephropathy can be particularly challenging. Similarly, relying on clinical assessment alone makes it difficult to distinguish whether a worsening of renal function encountered in a patient with an established diagnosis of lupus nephritis is caused by a flare of the disease or by the progression of chronic kidney damage. In such cases, renal biopsy is currently required to achieve a definitive diagnosis and to guide through an appropriate treatment.

However, this may not be necessary in the future. Perez et al. found that urinary exosomes miRNA 146a performed extremely well in distinguishing patients with SLE from those with a concomitant lupus nephritis with great sensitivity and specificity. Moreover, they found that among patients with LN miRNA-146a, levels were higher in the active LN group, enabling differentiation from those with inactive LN [30]. These findings underscore its potential as a non-invasive diagnostic tool in lupus nephritis. Data in the literature also found that urinary exosomes can perform well also as a prognostic marker; in fact, Garcia et al. found that an overexpression of miR-31, miR-107, and miR-135b-5p were associated with a better response to therapy [40].

- Paraprotein related nephropathies

Renal involvement in the context of plasma cell dyscrasia is a common finding. When a kidney biopsy cannot be performed, establishing a diagnosis can be particularly challenging, as a potential decline in renal function in a patient with a monoclonal gammopathy may be attributed to either the development of an MGRS or to unrelated factors. Alvarado et al. [31] demonstrated that patients with active amyloidosis exhibit a distinct urinary exosome profile, characterized by the presence of light chain decamers. This pattern differs from that observed in patients with LCDD, who predominantly show light chain tetramers. Notably, such multimeric forms were absent in patients with MGUS and other controls, suggesting a potential pathophysiological role for these higher-order light chain assemblies. They also found patients with AL amyloidosis can be distinguished from those with other monoclonal gammopathies by the presence of the so-called “amyloid signature”: a specific pattern of proteins, such as SAP, apoE, and vitronectin, expressed in urinary exosomes alongside the pathological light chain. This signature reflects the unique protein microenvironment associated with amyloid fibril formation and may serve as a non-invasive diagnostic marker.

- Rejection after kidney transplant

The diagnosis of allograft rejection in kidney transplant recipients is currently unavoidably dependent on renal biopsy. The histological examination remains the gold standard, as it allows the classification of rejection according to the Banff criteria and enables accurate prognostic stratification. However, the procedure carries significant risks, especially considering that the graft often represents the patient’s only functioning kidney. In such cases, vascular complications related to the biopsy may compromise the transplant outcome and lead to graft loss.

Beyond procedural risks, it is also important to consider that some patients are not eligible for renal biopsy due to conditions such as bleeding diathesis or ongoing double antiplatelet therapy. In these scenarios, interpreting a decline in graft function becomes particularly challenging. Potential causes—including infection, recurrence of the primary disease, or rejection—may present with overlapping clinical and laboratory features, making differential diagnosis difficult without histological confirmation.

Moreover, distinguishing between T cell-mediated rejection and antibody-mediated rejection, which require different therapeutic approaches, is especially complex without kidney biopsy. This underscores the critical need for alternative, non-invasive diagnostic strategies to support clinical decision-making in kidney transplant care.

A recent study by El Fakih et al. [32] analyzed the urinary exosomes of 192 patients with a kidney transplant biopsy and were able to find a different signature between those with all cause’s allograft rejection and the controls (patients without any sign of rejection) with a great negative predictive value. Moreover, they also found that there was an additional different pattern of expression in patients with antibody-mediated rejection (ABMR) and T cell-mediated rejection (TCMR). The signature that discriminates TCMR from ABMR showed some overlapping with the any-cause rejection signature and two additional genes (IFNAR2 and CD44) can help distinguish ABMR from TCMR.

- Diabetic Nephropathy

Diabetic nephropathy (DN) is commonly regarded as the leading cause of chronic kidney disease (CKD) worldwide; however, only a small fraction of patients with diabetes mellitus undergoes a biopsy-confirmed diagnosis of DN. Indeed, renal impairment in diabetic individuals may often stem from alternative etiologies—including immunologic, vascular, or hematologic disorders—and determining which patients warrant renal biopsy remains a clinical challenge. In this context, urinary exosomes have gained attention as a valuable non-invasive diagnostic adjunct. Notably, elevated levels of uromodulin (UMOD) in urinary exosomes have been detected in patients with DN prior to the onset of microalbuminuria, suggesting their utility as an early biomarker of disease onset [33]. Furthermore, a significant upregulation of miR-151a-3p, miR-192a-1-5p, let-7c-5p, and miR-182-5p has been observed in the urinary exosomes of patients with biopsy-proven DN compared to diabetic individuals without nephropathy, offering an additional layer of specificity for differential diagnosis [34,35]. Water channel aquaporins (AQPs), expressed at the plasma membrane of epithelial tubular cells, are often dysregulated during DN. Luigi Rossi et al. [36] analyzed the urine excretion of AQP5 and AQP2 (uAQP5 and uAQP2) via exosomes, finding a positive correlation with the histological class of DN. Collectively, these findings underscore the potential of urinary exosomal profiling to enhance diagnostic precision and guide clinical decision-making in the management of diabetic patients with renal involvement.

- Autosomal Dominant Polycystic Kidney Disease (ADPKD)

ADPKD is the most prevalent hereditary renal disease, characterized by the presence of multiple, bilateral kidney cysts and associated cysts in other organs, including liver, pancreas, and arachnoid membranes [41]. Two distinct genes have been identified as causative agents: PKD1 (chromosome 16p13.3) and PKD2 (4q21), both identified as key contributors to the structure and function of primary cilia. Proteomic analysis of urinary exosome-like vesicles in patients with ADPKD has revealed the presence of gene products related to the disease, suggesting that these vesicles may serve as PKD biomarkers [13]. Research conducted on patients diagnosed with ADPKD and PKD1 mutations has indicated a decrease in the levels of PC-1 and PC-2, and an increase in the levels of transmembrane protein 2 in urinary exosomes. Abnormal levels of cystin and ADP-ribosylation factor-like 6 have also been detected [42,43]. Furthermore, increased levels of complement proteins C3 and C9 were found in urinary extracellular vesicles from ADPKD patients, regardless of CKD status [44]. Conversely, elevated levels of envoplakin, periplakin, and villin-1 were exclusively observed in patients with progressive CKD [44], suggesting their potential utilization as biomarkers for disease progression.

## 6. Critical Appraisal of the Existing Literature

A closer inspection of the current literature reveals several key methodological limitations that compromise the robustness and generalizability of the results. First, the majority of studies are of a retrospective and/or observational nature. This methodological framework does not permit the establishment of causal relationships, thus giving rise to potential selection biases. Moreover, the limited sample size is a substantial limitation, as it diminishes statistical power and the potential for deriving definitive conclusions, necessitating a cautious interpretation of the results. Another recurring issue concerns the study population, which frequently originates from a single center or designated groups, thereby constraining the applicability of results to other populations or clinical contexts. Moreover, in many cases, the control groups were composed mainly of healthy subjects. This methodological choice does not allow for an in-depth evaluation of the specificity of the biomarkers analyzed, as there is no direct comparison with populations affected by other potentially confounding diseases. The limitations include an insufficient follow-up period to evaluate long-term outcomes and the absence of a comprehensive analysis of all potentially relevant clinical variables, which may have influenced the observed associations. In summary, the present narrative review underscores the necessity for prospective, multicentric studies with larger samples to corroborate the preliminary results and furnish more robust and generalizable evidence.

## 7. Therapeutic Applications

The properties of urinary exosomes make them optimal targeted therapeutic delivery vehicles. Their lipid bilayer structure protects their content and allows their selective fusion with recipient cells, enabling the direct intracellular delivery of their cargo. This targeted delivery mechanism permits modulation of specific molecular pathways within diseased or injured tissues, enhancing therapeutic precision and minimizing systemic side effects. Exosomes have been investigated as carriers of various therapeutic agents, including small interfering RNAs capable of crossing the blood–brain barrier [45], as well as bioactive compounds like the anti-inflammatory agent curcumin—which, when delivered to active monocytes, reduced inflammatory damage in a murine model of septic shock [46]—and chemotherapeutics such as doxorubicin, leading to high intratumoral accumulation of the drug with a reduced off-target exposure [47]. These studies highlight their versatility as carriers in a wide range of pathological contexts.

In the kidney specifically, compelling preclinical data indicate that exosome-mediated cargo can reprogram pathological pathways: in one study using a murine model of unilateral ureteral obstruction [48], mesenchymal stem cells (MSCs) were genetically modified to overexpress miR-let7c. The miRNA-enriched exosomes were found to significantly mitigate renal damage by reducing fibrotic progression, primarily through the downregulation of key profibrotic mediators such as collagen type IV α1, MMP-9, and TGF-β1. This demonstrates how exosomes can act not just as simple carriers, but also as potent epigenetic modulators.

Moreover, studies in models of renal ischemia–reperfusion injury (IRI), exosomes derived by bone marrow mesenchymal stem cells have demonstrated the ability to modulate inflammatory responses by influencing macrophage polarization. Xie et al. found that stem cell-derived exosomes could facilitate the shift of macrophages from a pro-inflammatory M1 state to an anti-inflammatory M2 state, causing a reduction in tissue inflammation and also encouraging regeneration [49].

The ability of exosomes to influence diverse signaling pathways and modulate the immune environment makes them promising tools for addressing complex diseases.

## 8. Conclusions

Urinary exosomes have emerged as a compelling frontier in nephrology. However, the huge potential of urinary exosome analysis is presently constrained by substantial technical and methodological limitations.

The reliance on sophisticated and often expensive instrumentation for their isolation and characterization represents one of the main limitations. Moreover, the absence of universally accepted protocols for exosome detection and analysis results in considerable heterogeneity across studies. This variability in methods makes it difficult to compare results across studies, which prevents the development of reliable and consistent datasets.

Despite these obstacles, the continued refinement and eventual standardization of exosome processing techniques hold immense promise. The development of harmonized protocols and the creation of shared datasets could dramatically enhance the clinical utility of urinary exosomes. Such advancements might enable the incorporation of exosomal profiling into routine nephrological practice as follows: augmenting differential diagnoses, providing surrogate tools for renal biopsy in selected contexts, and identifying prognostic indicators and biomarkers of therapeutic response, which are currently missing for many kidney diseases.

Moreover, urinary exosomes also represent an intriguing therapeutic platform thanks to their innate biocompatibility and ability to selectively deliver bioactive molecules to target cells.

In conclusion, further studies are needed to standardize detection methods and to confirm their clinical value in order to turn them into powerful tools, aiming for a new era of precision nephrology.

## Figures and Tables

**Table 1:** Urinary exosomal biomarkers associated with major nephropathies.

Nephropathy	Exosomes Biomarkers	References
IgA Nephropathy	(+) aminopeptidase N (+) Vasorin precursor (+) α-1-antitrypsin (+) ceruloplasmin (+) miR-29c (+) miR-146a (+) miR-205	[26] [26] [26] [26] [27] [27] [27]
Minimal Change Disease	(+) miR-1225-5p	[28]
Membranous Nephropathy	(+) Alix (+) CD63 (+) TSG10	[29] [29] [29]
Lupus Nephritis	(+) miR-146a	[30]
AL Amyloidosis	(+) SAP (+) apoE (+) vitronectin (+) light chain decamers	[31] [31] [31] [31]
All cause rejection	(+) (CXCL11, CD74, IL32, STAT1, CXCL14, SERPINA1, B2M, C3, PYCARD, BMP7, TBP, NAMPT, IFNGR1, IRAK2)	[32]
ABMR	(+) (CD74, C3, CXCL11, CD44, IFNAR2)	[32]
Diabetic Nephropathy	(+) uromodulin (+) miR-151a-3p (+) miR-182-5p (+) let-7c-5p (+) miR-192a-1-5p AQP5, AQP2	[33] [34] [34] [34] [35] [36]

(+) = Overexpression.

## Abbreviations

The following abbreviations are used in this manuscript:

ABMR	Antibody Mediated Rejection
ADP	Adenosine Diphosphate
ADPKD	Autosomal Dominant Polycystic Kidney Disease
AF4	Asymmetric Flow Field-Flow Fractionation
apoE	Apolipoprotein E
AQP	Aquaporin
ATPase	Adenosine Triphosphatase
CD	Cluster of Differentiation
CKD	Chronic Kidney Disease
DN	Diabetic Nephropathy
ESCRT	Endosomal Sorting Complex Required for Transport
ESDR	End-Stage Renal Disease
EV	Extracellular Vesicle
EXODUS	Exosome detection via the ultrafast isolation system
FSGS	Focal Segmental Glomerulosclerosis
GFR	Glomerular Filtration Rate
IFNAR2	Interferon-Alpha/Beta Receptor Subunit 2
IgA	Immunoglobulin A
IgAN	Immunoglobulin A Nephropathy
IgG	Immunoglobulin G
ILV	Intraluminal Vesicle
ISEV	International Society for Extracellular Vesicles
LCDD	Light Chain Deposition Disease
LN	Lupus Nephritis
MCD	Minimal Change Disease
MGRS	Monoclonal Gammopathy of Renal Significance
MGUS	Monoclonal Gammopathy of Undetermined Significance
miR/miRNA	Micro Ribonucleic Acid
MMP-9	Matrix Metalloproteinase-9
mRNA	Messenger Ribonucleic Acid
MSC	Mesenchymal Stem Cell
MVB	Multivesicular Body
NLR	Nucleotide-binding Leucine-rich repeat Receptors
NLRP3	NLR Family Pyrin Domain Containing 3
NRF2	Nuclear factor erythroid 2-related factor 2
PEG	Polyethylene Glycol
PLA2R	Phospholipase A2 Receptor, M-type
SAP	Serum Amyloid P component
SLE	Systemic Lupus Erythematosus
TBMN	Thin Basement Membrane Nephropathy
TCMR	T-Cell Mediated Rejection
TGF-β	Transforming Growth Factor Beta
THSD7A	Thrombospondin Type-1 Domain-Containing 7A
TSG101	Tumor Susceptibility Gene 101
uAQP	Urinary Aquaporin
UMOD	Uromodulin
VPS4	Vacuolar Protein Sorting-associated protein 4
